# New Surgical Technique for Robotic Myomectomy: Continuous Locking Suture on Myoma (LSOM) Technique

**DOI:** 10.3390/jcm10040654

**Published:** 2021-02-08

**Authors:** Sa Ra Lee, Eun Sil Lee, Hye Rim Eum, Young-Jae Lee, Shin-Wha Lee, Jeong Yeol Park, Dae-Shik Suh, Dae-Yeon Kim, Sung Hoon Kim, Yong-Man Kim, Young-Tak Kim

**Affiliations:** 1 Department of Obstetrics and Gynecology, University of Ulsan College of Medicine, Seoul Asan Medical Center, 88, Olympic-ro 43-gil, Songpa-gu, Seoul 05505, Korea; hye8383@naver.com (H.R.E.); lyjobgy@gmail.com (Y.-J.L.); swhlee@amc.seoul.kr (S.-W.L.); catgut1-0@hanmail.net (J.Y.P.); dssuh@amc.seoul.kr (D.-S.S.); kdyog@amc.seoul.kr (D.-Y.K.); kimsung@amc.seoul.kr (S.H.K.); ymkim@amc.seoul.kr (Y.-M.K.); ytkim@amc.seoul.kr (Y.-T.K.); 2 Department of Obstetrics and Gynecology, Soonchunhyang University Seoul Hospital, Soonchunhyang University College of Medicine, Seoul 04401, Korea; eslee165@schmc.ac.kr

**Keywords:** cost, myomectomy, robot-assisted laparoscopic myomectomy, locking suture on myoma technique

## Abstract

Robot-assisted laparoscopic myomectomy (RALM) has broadened the indications even in complex myomas. However, the high cost of RALM remains the main disadvantage. Therefore, a surgical technique that can reduce the cost of RALM and still has the advantages of robotic surgery is required. We propose a “locking suture on myoma (LSOM)” technique and compared the operative and perioperative outcomes of patients who underwent RALM with or without the LSOM technique. We included 337 patients who underwent RALM with (*n* = 160) or without (*n* = 177) the LSOM technique between March 2019 and August 2020. The LSOM group had low parity and gravidity, with a low rate of Cesarean sections. Myoma type was not different between the groups; however, patients in the LSOM group had larger, heavier, and higher number of myomas, although fewer patients had multiple myomas and were discharged earlier. Total operating time, estimated blood loss, pre- and postoperative hemoglobin levels, transfusion rate, and postoperative fever were not different between the two groups. In conclusion, the LSOM technique may be a viable surgical option for myomas, as it can reduce the cost of RALM by obviating the need for robotic Tenaculum forceps.

## 1. Introduction

Uterine myoma is a common benign gynecologic tumor in reproductive-aged women, and myomectomy is the standard fertility-preserving surgical option [1,2]. The use of minimally invasive surgery, including laparoscopic myomectomy and robot-assisted laparoscopic myomectomy (RALM), is growing rapidly [3,4,5,6]. However, laparoscopic myomectomy has a limitation in multiple-layered intracorporeal myometrial suturing that is often challenging even for experienced laparoscopic surgeons. Robotic surgical systems can help overcome this obstacle by EndoWrist technology (Intuitive Surgical, Sunnyvale, CA, USA), which allows the articulation of instruments by up to 540° and 45° for multi-port and single-site robotic instruments, respectively. Therefore, RALM has an advantage for easier multiple intracorporeal suturing than laparoscopic myomectomy.

However, the high cost of RALM is a major disadvantage [4,5,6,7,8]. Varghese et al. [9] reported the effectiveness and costs of hysterectomy: the cost of robotic hysterectomies was 1.5–3 times higher than the costs of conventional techniques, including laparoscopic or vaginal approaches [9]. Tapper et al. [10] also showed that the cost of RALM was consistently higher than the cost of abdominal myomectomy or laparoscopic myomectomy, unless the robotic disposable equipment costs were less than $1400. However, another study in the US concluded that there was no cost difference between RALM and laparoscopic myomectomy when performed by surgeons who were past their initial learning curve [11]. In South Korea, the cost of RALM is typically between $6000 and $10,000, which is 2–4 times higher than the cost of laparoscopic myomectomy [6]. The cost for a robotic surgery consists of a fixed cost for purchasing the robotic surgical system and disposable equipment cost per case [12]. The expense of robotic surgery increases proportionally to the number of robotic instruments used. Therefore, there is a need for a surgical technique that can reduce the number of instruments used per robotic surgical procedure while retaining the merits of a robotic surgery.

The robotic instruments frequently used in RALM are Fenestrated or Maryland bipolar forceps, monopolar scissors or a hook bovie, and a needle driver. Robotic Tenaculum forceps can also be used in multi-port RALM for traction of myomas during myoma enucleation. Our proposed locking suture on myoma (LSOM) technique can obviate the need for robotic Tenaculum forceps and, therefore, can decrease the cost of RALM. The LSOM technique requires only a few barbed suture materials, such as 1-0 V-Loc (Covidien Medtronic, Minneapolis, MN, USA), which can enable the reduction of RALM expenses by nearly $400, the cost spent for the use of robotic Tenaculum forceps during surgeries in South Korea.

Moreover, we can expect a little benefit in terms of blood loss with this LSOM technique. Because surgeons usually encounter heavy bleeding from the exposure of well-perfused normal myometrial tissues just before the complete detachment of the myoma from the uterus, mainly from the vessels at the base of the myoma, rapid myometrial suturing just after detaching the myoma from the uterus is significant to decrease the blood loss during myomectomy [13]. Surgeons can remove multiple myomas with minimal time delay from the time of myoma removal to myometrial suturing, which may reduce the estimated blood loss (EBL) [14,15,16].

In addition to the above-mentioned possible advantages, this technique can reduce the operating time (OT) by obviating the time for finding the removed myomas in the pelvic cavity. Minimally invasive myomectomy for multiple myomas often requires extra time for identifying the retrieved myomas in the abdominal cavity. Surgeons usually remove the retrieved myomas at the end stage of the minimally invasive myomectomy by intracorporeal or extracorporeal morcellation via an intra-abdominal uncontained power morcellation or a protected in-bag extracorporeal manual morcellation [17,18], instead of removing the retrieved myomas piece-by-piece whenever the myomas were removed from the uterus, as in abdominal myomectomies. However, this surgical procedure can sometimes be challenging, as the retrieved myomas can become lost in the abdominal cavity and embed within the bowel loop. This state increases the overall OT. To prevent this, several surgeons use a laparoscopic specimen retrieval bag and place the retrieved myomas into the bag during the surgery. After the surgery, the collected myomas in the retrieval bag are easily removed. However, the myomas can sometimes escape from the retrieval bag during surgery and embed within the bowel loop. With the LSOM technique, surgeons can align the retrieved myomas and collect them all at once by grasping the thread sutured on the myomas. Several surgeons have used the method of suturing retrieved myomas one-by-one and lining them up, creating a lace of myomas similar to a pearl necklace, and prevent the escape of retrieved myomas into the pelvic cavity [16]. However, the LSOM technique is different in that it creates consecutive step-by-step locking sutures on myomas as soon as they are exposed after a uterine incision before retrieval, which not only prevents myoma loss but also adds easy traction without the need to use robotic or laparoscopic Tenaculum forceps.

Therefore, in this study, we compared RALM cases with or without the LSOM technique to evaluate the feasibility of the said technique and possibility of replacing the robotic instrument, Tenaculum forceps. Furthermore, we aimed to evaluate the LSOM technique in terms of EBL and OT.

## 2. Materials and Methods

### 2.1. Study Design and Patients

In this retrospective study, we included all patients undergoing RALM between 1 March 2019 and 31 August 2020 at the Seoul Asan Hospital. The type of surgery (RALM, laparoscopic myomectomy, or abdominal myomectomy) was decided after counseling patients regarding the size and number of myomas, and based on future pregnancy plans, desire in preserving the uterus, the patient’s preference, and cost. The LSOM technique was initially developed in 2017 [19] by one surgeon (Lee SR); RALM procedures that did not use the LSOM technique were performed by fellowship-trained robotic surgeons who completed at least 15 cases of RALM.

We obtained the following data from each patient’s chart review: age, gravidity, parity, body mass index (BMI), and history of previous abdominal surgery, including Cesarean section. The diameter of the dominant myoma was measured on preoperative ultrasound images. Preoperative determination of size and location of myomas is crucial before minimally invasive myomectomy. This is especially important for multiple myomas or when preoperative work-up is based solely on pelvic ultrasonography, which may underestimate the number of myomas [20,21]. We opted for magnetic resonance imaging in 152 cases (45.1%) wherein we there was a need to differentiate between adenomyoma and malignancy or to perform ensure a more accurate assessment in cases with multiple myomas or large myomas adjacent to the uterine endometrium. Myomas were categorized into the International Federation of Gynecology and Obstetrics (FIGO) systems, type 0–3 (submucosal), 4–5 (intramural), 5–6 (subserosal), 7 (pedunculated subserosal) or 8 (intraligamentary, cervical, and parasitic) myomas [22].

We also obtained the following surgery-related data: use of LSOM technique, concomitant surgery, total operative time from skin incision to closure (as measured by the anesthesiologist intraoperatively), EBL, number of removed myomas, conversion to laparotomy, and blood transfusion. Data on perioperative outcomes, including the weight of the removed myomas (as weighed by the pathologist during pathologic examination), length of hospital stay, postoperative fever within 48 h, and any complications related with RALM, were also collected.

### 2.2. Surgical Procedures

RALM was performed using the da Vinci Si and Xi system with central or side docking types. In the case of multi-port RALM, under general anesthesia, a 2.5 cm intraumbilical incision was made and a glove port (Sejong Medical, Paju, Gyeonggi-do, Korea) was inserted. Two 8 mm skin incisions were made at the left and right sides of the umbilical incision for two robotic trocars. Robotic instruments, a monopolar spatula bovie (permanent cautery) or monopolar scissors for the right robotic arm, and fenestrated bipolar forceps for the left robotic arm were used for the uterine incision procedure. The right robotic arm was replaced with a Mega Suture Needle Driver^®^ during the closure of the uterine incision, which is during myometrial suturing.

In the case of single-site RALM, a vertical incision of approximately 2.5–2.7 cm was made in the umbilicus, and the glove port (Sejong Medical) was inserted. Semirigid robotic instruments, a monopolar hook bovie (permanent cautery) for the right robotic arm, and fenestrated bipolar forceps for the left robotic arm were used for the uterine incision procedure. An oblique uterine incision was made for the perpendicular myometrial suturing in the 2 to 8 o’clock direction considering the range of the angle of the wristed needle driver.

**Figure 1:**
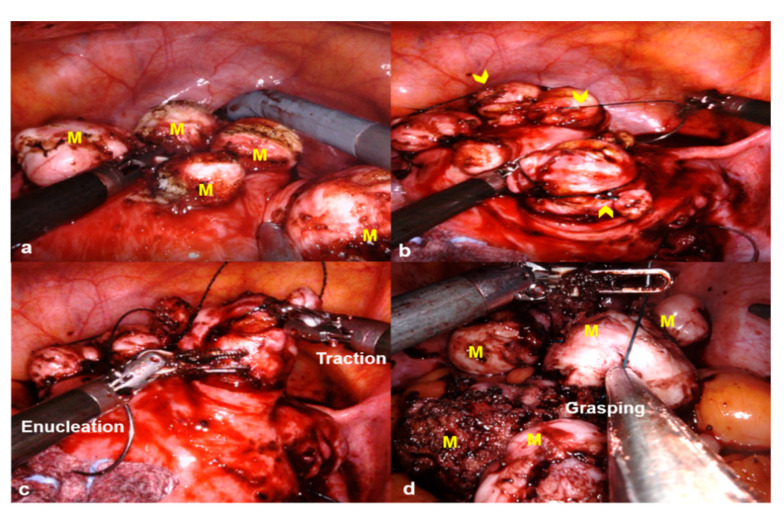
Locking suture on myoma (LSOM) technique. (**a**) Multiple myomas (M) are exposed after an incision on the uterine wall. (**b**) Locking sutures with 1-0 V-Loc (arrowheads) are made on the exposed uterine myomas. (**c**) During the myoma enucleation process, the myoma traction is easily performed by grasping the thread with a mega suture needle holder. (**d**) The retrieved myomas are easily removed by simply grasping the thread with a laparoscopic needle holder.

For the LSOM technique, upon exposure of the myoma (Figure 1a), a locking suture with 1-0 V-Loc was made on the uterine myoma (Figure 1b), and the myoma traction was easily performed by grasping the thread with the fenestrated bipolar forceps, needle holder, or laparoscopic atraumatic forceps (Figure 1c). Whenever the dissection between the myoma and myometrium was advanced, the next locking suture was made on the additionally exposed myoma tissue at the nearest position to the dissection plane, and this process was repeated. This step-by-step locking suture offered an easier enucleation of the myoma than using the laparoscopic Tenaculum forceps. After the complete separation of the myoma from the uterus, monopolar or bipolar electrocauterization was applied for hemostasis. The uterine incision was closed with a two- or three-layered closure using the 1-0 V-Loc in a continuous running suture technique. The serosal layer was sutured with a baseball or interlocking suture technique using the 1-0 V-Loc or 1-0 or 2-0 Quill™ SRS bidirectional barbed suture (Angiotech Pharmaceuticals, Inc., Vancouver, BC, Canada). The retrieved myomas were easily removed by simply grasping the thread with laparoscopic atraumatic forceps or laparoscopic needle holder (Figure 1d) and were extracted by manual morcellation using a scalpel within a bag, or sometimes in an uncontained state through the existing umbilical incision. After the removal of myomas, we thoroughly examine the pelvic cavity without changing the patient’s position to check for spillage of myoma tissue. For all cases, there were no additional skin incisions, converting to laparotomy or hybrid RALM involving minilaparotomy for tissue extraction, as demonstrated by Gingold et al. [14].

### 2.3. Statistical Analysis

To compare continuous variables between the LSOM and non-LSOM groups, we used the Student’s *T*-test. To compare the proportions of categorical variables between the two groups, we used the chi-square test. The data were normally distributed (*p* > 0.05, Kolmogorov–Smirnov test). In addition to single-variable analysis, multiple linear regression analysis was used to identify the factors that were significantly related to EBL in the LSOM and RALM groups after adjusting for possible confounding factors which can be associated with blood loss. We performed a statistical analysis of 16 single variables (type of myoma, maximal myoma diameter, weight of the removed myomas, number of removed myomas, multiple myomas, number of large myomas over 3 cm, total operative time, EBL, pre- and postoperative hemoglobin (Hb) levels, changes between pre- and postoperative Hb levels, transfusion rate, number of transfused packs, length of hospital stay, and perioperative complications) to determine whether the observed values of these variables were significantly different between the LSOM and non-LSOM groups. Since there were several variables in the test, we applied the Bonferroni’s multiple testing correction to avoid false-positive results. The cut-off *p* value for significance was 0.05/16 = 0.003.

All computations were performed with R, a language and environment for statistical computation (R Foundation for Statistical Computing, Vienna, Austria) [23].

## 3. Results

### 3.1. Patient Baseline Characteristics

In total, 160 cases of RALM using the LSOM technique, LSOM group (160 cases, multi-port, *n* = 135; single-site, *n* = 25) and 177 cases not using the LSOM technique, non-LSOM group (177 cases, multi-port, *n* = 176; single-site, *n* = 1) were included in this study. Patient age, BMI, and history of pelvic surgery other than cesarean section did not significantly differ between the LSOM and non-LSOM groups (*p* > 0.05). The LSOM group had low parity and gravidity, with a low rate of Cesarean section history (*p* < 0.05; Table 1). The type of myoma was not different between groups; however, the LSOM group (9.20 ± 3.17 cm) had a larger mean maximal diameter of the dominant myoma than the non-LSOM group (7.94 ± 2.49 cm; *p* < 0.05). The LSOM group (135 cases (84.4%)) had fewer cases of multiple myomas than the non-LSOM group (176 cases (99.4%)); however, the LSOM group had more myomas over 3 cm (median = 1, range (1–10)) than the non-LSOM group (median = 1, range (1–6); *p* < 0.05). The mean weight of the removed myomas was available in 120 LSOM group cases and 136 non-LSOM group cases and was significantly heavier in the LSOM group (359.52 ± 313.44 (6–1425) g vs. 241.88 ± 202.10 (10–1352) g, respectively; Table 2).

### 3.2. Comparison of Single Variables between the LSOM and Non-LSOM Groups

Among the 16 variables, six showed significant differences in their means or proportions between the LSOM and non-LSOM groups. The variables that had significant results were maximal myoma diameter, number of removed myomas, multiple myomas, number of myomas over 3 cm, weight of the removed myomas, and length of hospital stay. The number of removed myomas and the number of large myomas over 3 cm showed the most significant result (*p* < 2.2 × 10^−16^), followed by the length of hospital stay (*p* = 2.75 × 10^−16^). However, the total operative time was not different between the LSOM and non-LSOM groups (141.43 ± 72.47 min vs. 147.71 ± 58.06 min, *p* = 0.38). While the EBL, preoperative Hb level, and changes between pre- and postoperative Hb levels were nominally significant (*p* < 0.05), their *P* values were higher than the adjusted *P* values (0.003); therefore, the results were not significant. The LSOM group (*n* = 2, range (2–22)) had a significantly shorter mean length of hospital stay than the non-LSOM group (*n* = 3, range (0–8); Table 2).

### 3.3. Results of Multiple Linear Regression Analysis

We selected variables possibly associated with EBL based on the results of the single-variable analysis and our previous knowledge. Variables included age, BMI, history of pelvic surgery, concomitant surgery, type of myoma, maximal myoma diameter, number of myomas (>3 cm), weight of the removed myomas, total operative time, and type of surgery (LSOM or non-LSOM). There were no factors associated with EBL, including LSOM (Table 3).

## 4. Discussion

### 4.1. Feasibility of the LSOM Technique in RALM

We demonstrated that our proposed LSOM technique enabled RALM to be feasible for larger, heavier, and a greater number of myomas than non-LSOM without the use of robotic Tenaculum forceps, within a similar OT. Patients who underwent the RALM with LSOM technique were discharged earlier than those who underwent RALM without the LSOM technique, with similar EBL and pre- and postoperative Hb changes.

In the multiple linear regression analysis, EBL was not associated with any of the investigated factors; therefore, we could not demonstrate the effect of LSOM on EBL. However, we demonstrated that RALM could be successfully performed without conversion to abdominal myomectomy even in large, heavy, multiple myomas without robotic Tenaculum forceps by using the LSOM technique, a very simple technique that can decrease the cost of RALM.

Our hypothesis was that the LSOM technique can decrease blood loss or transfusion; however, our study failed to demonstrate this. Several authors have developed surgical techniques to minimize blood loss during myomectomy, although only a few have been shown to reduce the need for transfusion, and not all studies were in laparoscopic myomectomy [23,24,25,26,27]. Naval et al. [16] suggested several tips of laparoscopic myomectomy for multiple myomas, such as intermittent use of vasopressin, suturing using barbed sutures, and creating a lace and garland of myomas. A Cochrane review published in 2014 analyzed interventions to reduce hemorrhage during myomectomy and found that bupivacaine with epinephrine, tranexamic acid, gelatin-thrombin matrix, a pericervical tourniquet, ascorbic acid, dinoprostone, loop ligation, and a fibrin sealant patch may reduce bleeding during myomectomy [23]. Morcellation in a bag prevents the dissemination of bits of myoma and visceral injury [28,29,30]. A prior study investigated a myoma enucleation technique by morcellation while it was still attached to the uterus in 44 patients with at least one myoma over 7 cm and/or presence of three or more myomas over 5 cm in size, similar to our study design. The technique was found to be safe and efficient; however, it did not result in a decreased blood loss [17].

In contrast to our hypothesis, the LSOM technique did not decrease the OT. However, considering that the LSOM group had larger myomas, a higher number of myomas with more myomas over 3 cm, and heavier myomas than the non-LSOM group, further analysis of matched case-controls or propensity score matching will be needed to draw an evident conclusion.

In terms of the effect of the LSOM technique in preventing the loss of retrieved myomas, the event of losing myomas was not recorded in the operation records in our retrospective study design. Therefore, a prospective study and long-term follow-up data for the occurrence of parasitic myomas after RALM will be needed to conclude this effect.

### 4.2. Application of LSOM Technique in Single Incision RALM

**Figure 2:**
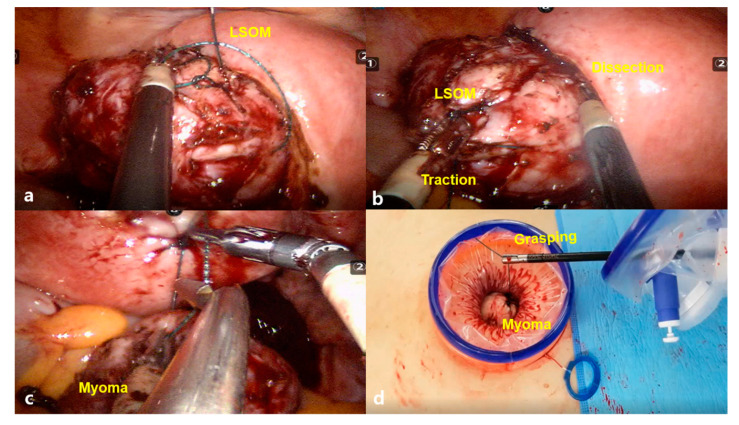
The locking suture on myoma (LSOM) technique in single-site robotic myomectomy. (**a**) Traction of myoma is performed by grasping the thread with a semirigid fenestrated bipolar forceps, and the next suture for the LSOM technique is applied. (**b**) During the myoma enucleation process, the traction of myoma becomes easy by grasping the thread with a semirigid fenestrated bipolar forceps (**c**). (**d**) The retrieved myoma can be easily removed by simply grasping the thread with any instruments, such as robotic wristed needle holder, laparoscopic needle holder, or atraumatic forceps.

This technique can be especially useful in single-site or single-port (SP) RALM (Figure 2), as there are no available semirigid robotic Tenaculum forceps for single-site or SP robotic surgery systems.

**Figure 3:**
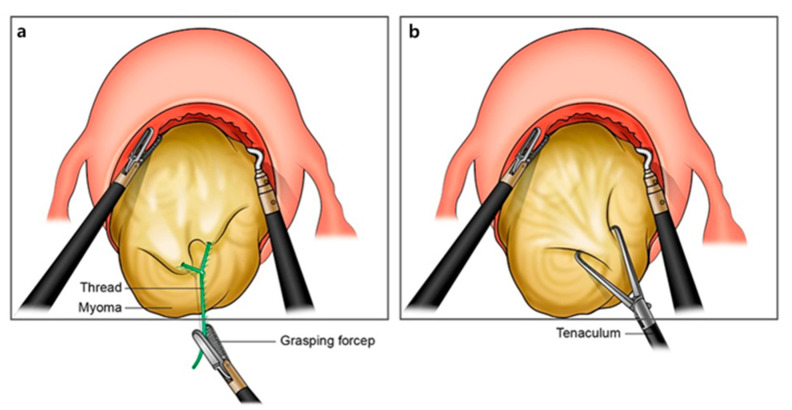
Comparison of the two traction methods: (**a**) locking suture on myoma (LSOM) technique and (**b**) conventional method using Tenaculum forceps.

We can use the monopolar hook for traction in RALM. However, it has a weak traction strength compared with the Tenaculum forceps for multi-port RALM or laparoscopic myomectomy, and this is a cause of the long OT in single-site RALM [31,32]. We should insert a conventional laparoscopic Tenaculum forceps held by an assistant into the single intraumbilical port for the traction of the myoma during enucleation. In this case, we commonly encounter overcrowding among the semirigid robotic instruments, camera, and assistant’s conventional laparoscopic instruments. Despite the single-site robotic system was developed to reduce the overcrowding between instruments that was commonly encountered in conventional single-port laparoscopic surgeries, the challenge has not been completely solved. The LSOM technique can partially resolve this difficulty by replacing the space occupied by the 5 mm laparoscopic Tenaculum forceps with thinner threads of V-Loc, providing a more spacious umbilical port, as illustrated in Figure 3.

### 4.3. LSOM Technique for Safe Minimally Invasive Myomectomy

As parasitic myomas can develop after myomectomy, the LSOM technique might decrease this risk by preventing the loss of retrieved myomas in the pelvic cavity. Even in cases of losing the retrieved myomas in the abdominal cavity, the myomas can be easily discovered through *X*-ray by recognizing the needle on the thread sutured in the myomas. Parasitic myomas may be encountered during emergency operations, such as diagnostic laparoscopies for abdominal pain, and during routine operations, such as appendectomies. Therefore, diagnostic emergency laparoscopies are often necessary for definite diagnosis and concomitant laparoscopic removal of parasitic myomas [33].

**Figure 4:**
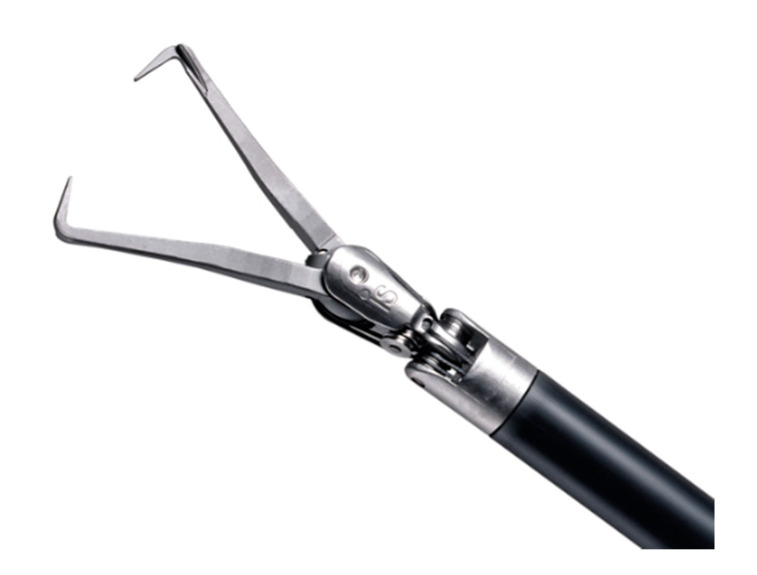
The sharp and powerful long jaw of the robotic Tenaculum forceps (© (2020) Intuitive Surgical, Inc. Used with permission) [34].

Furthermore, the LSOM technique can offer an easier modulation of force than the powerful robotic Tenaculum forceps. This can reduce not only the risk of entry into the uterine cavity, but also injury to the endometrium. In addition, surgeons should take special caution when they use the robotic Tenaculum forceps to not injure the adjacent organs, bowel, bladder, or peritoneum by the sharp and powerful long jaw of the forceps (Figure 4). Replacing the robotic Tenaculum forceps with the LSOM technique can reduce this risk. However, a randomized controlled trial is needed to validate the above-mentioned hypothesis regarding the effects of the LSOM technique.

### 4.4. Strengths and Limitations

This study has several strengths. First, to the best of our knowledge, this is the first study to compare RALM with or without the LSOM technique. Second, the relatively short study period of 18 months used in our study can preclude intrapersonal variation in surgical experiences. Third, the number of patients in each group was comparable (160 LSOM cases vs. 177 non-LSOM cases). Fourth, none of the cases required an additional skin incision or mini-laparotomy for tissue extraction and maintained the cosmetic benefit of minimally invasive surgery. Finally, this is a very simple, easy, and cost-effective surgical technique that can be readily performed by unexperienced surgeons for not only RALM but also laparoscopic myomectomy.

However, this study also had some limitations that should be considered. First, this was not a randomized controlled study. Second, there was a lack of data regarding the preoperative use of a gonadotropin-releasing hormone agonist or selective progesterone receptor modulator, history of uterine artery embolization, and use of perioperative medical agents to decrease blood loss, such as tranexamic acid, misoprostol, vasopressin, or oxytocin. However, all these factors cannot be adjusted in previous retrospective studies. Finally, the LSOM technique was used only by a single surgeon; thus, we cannot exclude selection bias. The characteristics of the non-LSOM group were not identical to the RALM group that used robotic Tenaculum forceps, which meant that all the cases in the non-LSOM group did not use the robotic Tenaculum forceps. Therefore, the comparison between the LSOM and non-LSOM groups may not be a direct comparison between the LSOM technique and the use of robotic Tenaculum forceps. Further studies of myomas similar in size, number, and location are required to draw a robust conclusion regarding the benefits of LSOM. This study’s results cannot be interpreted as LSOM could be preferable in case of large and heavy myomas. Suture was performed using barbed suture materials in all cases. Although we did not investigate pregnancy outcomes, recent studies on the use of barbed sutures during laparoscopic myomectomy have shown the impact on reproductive outcomes is similar to that achieved using smooth conventional threads [35,36]; therefore, we expect similar pregnancy outcomes following RM using barbed suture materials.

Although the only advantage of the LSOM technique compared with the robotic Tenaculum forceps demonstrated in this study was the reduction of cost, the possible advantages of the LSOM technique suggested above still warrant an opportunity to be evaluated in the future because negative effects were not demonstrated in this study.

## 5. Conclusions

The proposed LSOM technique of making step-by-step locking sutures on a myoma is a very simple technique that enables comfortable traction, localization, and retrieval of the myoma during RALM and can also be applied to laparoscopic myomectomy.

## Figures and Tables

**Table 1:** Patient baseline characteristics.

Characteristics	LSOM (*n* = 160)	Non-LSOM (*n* = 177)	*p*-Value
Age (years, mean ± SD)	37.23 ± 6.17	38.07 ± 5.89	0.21
BMI (kg/m^2^, mean ± SD)	23.03 ± 4.04	23.46 ± 4.20	0.86
Parity (median (range))	0 (0–3)	0 (0–3)	<0.001
Gravidity (median (range))	0 (0–7)	0 (0–6)	<0.001
Previous pelvic surgery			
Cesarean section, *n* (%)	6 (3.8%)	20 (11.4%)	0.02
Other pelvic surgery, *n* (%)	20 (12.5%)	24 (13.6%)	0.90

LSOM, locking suture on myoma; SD, standard deviation; BMI, body mass index.

**Table 2:** Comparison of operative and perioperative outcomes.

Outcomes	LSOM (*n* = 160)	Non-LSOM (*n* = 177)	*p*-Value
Type of main myoma *			0.26
Intramural (FIGO type 3, 4)	114 (71.25)	124 (70.05)	
Subserosal (FIGO type 5, 6)	30 (18.75)	34 (19.20)	
Pedunculated subserosal (FIGO type 7)	1 (0.62)	12 (14.12)	
Submucosal (FIGO type 0–2)	6 (3.75)	4 (2.25)	
Intraligamentary (FIGO type 8)	7 (4.37)	3 (1.69)	
Cervical (FIGO type 8)	1 (0.62)	0	
Parasitic (FIGO type 8)	1 (0.62)	0	
Maximal myoma diameter (cm, mean ± SD)	9.20 ± 3.17	7.94 ± 2.49	<0.001
Number of removed myomas (median (range))	2 (1–34)	2 (1–10)	<0.001
Multiple myomas	135 (84.4%)	176 (99.4%)	<0.001
Number of myomas >3 cm (median (range))	1 (1–10)	1 (1–6)	<0.001
Weight of the removed myomas (g, mean ± SD) ^†^	359.52 ± 313.44	241.88 ± 202.10	<0.001
Total operative time, (min, mean ± SD) ^‡^	141.43 ± 72.47	147.71 ± 58.06	0.38
Concomitant surgery	6 (3.8%)	20 (11.4%)	0.02
Estimated blood loss (mL, mean ± SD)	411.78 ± 806.64	241.16 ± 222.42	0.01
Preoperative Hb (g/dL)	12.64 ± 1.36	12.34 ± 1.40	0.04
Postoperative Hb (g/dL)	9.48 ± 1.66	9.56 ± 1.63	0.66
Difference in Hb (g/dL)	3.16 ± 1.69	2.78 ± 1.58	0.03
Transfusion	27 (25.8%)	35 (35.1%)	0.59
Number of transfused packs (median (range))	0 (0–17)	0 (0–5)	0.42
Length of hospital stay (days, mean ± SD)	2 (2–22)	3 (0–8)	<0.001
Postoperative fever (within 48 h)	21 (13.1%)	12 (6.8%)	0.08

Data are *n* ± SD, *n* (range), or *n* (%) unless otherwise stated. * FIGO, leiomyoma subclassification system [22]. ^†^ Data were available from 120 cases of LSOM and 136 cases of non-LSOM. ^‡^ Time from skin incision to skin closure. LSOM, locking suture on myoma: SD, standard deviation: Hb, hemoglobin: FIGO, the International Federation of Gynecology and Obstetrics.

**Table 3:** Factors associated with estimated blood loss.

Factors	Coefficient (95% CI)	*p* Value
Age	−7.41 (−21.57~6.76)	0.31
BMI	1.34 (−21.96~24.65)	0.91
History of pelvic surgery	−35.43 (−295.96~225.10)	0.79
Concomitant surgery	−102.01 (−428.03~224.01)	0.54
Multiple myomas	−152.35 (−485.80~181.09)	0.37
Maximal myoma diameter	−12.36 (−51.14~26.41)	0.53
Number of myomas	6.24 (−21.32~33.81)	0.66
Number of myomas >3 cm	−13.58 (−90.24~63.07)	0.73
Weight of the removed myomas	0.37 (−0.07~0.82)	0.10
Total operative time	0.52 (−0.82~1.87)	0.45
Type of surgery *	165.13 (−17.64~347.91)	0.08

* Type of surgery means locking suture on myoma (LSOM) or non-LSOM. CI, confidence interval; BMI, body mass index; EBL, estimated blood loss.

## Data Availability

The excel data used to support the findings of this study were supplied by Sa Ra Lee under license, and requests for access to these data should be made to S.R.L.
